# Pro - inflammatory cytokines and metalloproteinase activation in polypropylene mesh implant in rat subcutaneous tissue

**DOI:** 10.1590/S1677-5538.IBJU.2016.0553

**Published:** 2018

**Authors:** Elaine Bronzatto, Cássio Luis Zanettini Riccetto

**Affiliations:** 1 Departamento de Urologia, Universidade Estadual de Campinas – Unicamp, Campinas, SP, Brasil

**Keywords:** Metalloproteases, Cytokines, Polypropylenes, polyamide mesh [Supplementary Concept]

## Abstract

**Aims and Objectives:**

Polypropylene meshes have been increasingly adopted for correction of pelvic organ prolapse due to its lower recurrence rate when compared to surgeries without meshes. The study of the interaction of these materials with the host tissue may contribute to the development of materials with best biocompatibility and, consequently, less complication rates.

**Materials and Methods:**

The present study compares the inflammatory reaction of standard-weight (SW) and lightweight (LW) meshes (72 g/m^2^16g/m^2^ respectively), implanted in the abdomen of 20 adult rats, which were euthanized in four or 30 days. Quantification of pro-inflammatory markers, IL-1 and TNF-α, and of metalloproteinases, MMP2 and MMP3, were carried out through immunohistochemistry with AxioVision^®^ software.

**Results:**

There were no significant differences in the quantification of IL-1 and TNF-α in LW versus SW meshes. However, IL-1 quantification increased along time (30 days >4 days, p=0.0269). Also, MMP-2 quantification was similar to SW and LW and both presented a significant increase along time (30 days >4 days, p <0.0001). MMP-3 quantification also showed no difference between the SW and LW groups, but increased along time (30 days >4 days, p=0.02).

**Conclusions:**

Mesh's density did not influence the quantification of pro-inflammatory cytokines IL-1 and TNF-α and metalloproteinases 2 and 3. The increased expression of IL-1, MMP-2 and MMP-3 over time could represent a longstanding inflammatory response after PP mesh implantation. Possibly, the occurrence of adverse events following PP prosthetic implants can be influenced by other factors, not solely related to the amount of implanted material.

## INTRODUCTION

The use of meshes for pelvic organ prolapse (POP) correction and urinary incontinence has been widely adopted due to its efficacy, especially regarding recurrences, which are significantly lower than conventional techniques which are based on use of structurally compromised tissue. (1–5). In fact, POP recurrence after the advent of mesh implants have dropped from 30-50% to 10-30% (5).

However, complications related to compatibility flaws on tissue integration of the implanted material are still challenging despite the evolution of biomaterials. These complications, such as mesh erosion, infection and local pain directly affect quality of life, which has led the FDA (Food and Drug Administration) to recently publish warnings about the lack of class I proof of efficacy and safety on the use of meshes to correct POP (6). Thus, complications related to mesh tissue integration justify studies aiming at better understanding the inflammatory reactions that may compromise the healing process (7).

The weight of the mesh may influence the intensity of the inflammatory reaction and consequently the result of the surgery. There is no consensus in the literature on the classification of mesh weight, but using the classification of Coda et al., it is considered lightweight <70 g/m^2^, standard-weight ≥70<140 g/m^2^ and heavy weight >140 g/m^2^ (8).

Surgery, as well as a mesh implantation, generates a local wound, which triggers the release of cytokines and growth factors such as interleukin-1 (IL-1), transforming growth factor beta (TGF-β) and tumor necrosis factor alpha (TNF-α). In the presence of such substances, neutrophils, macrophages, monocytes, fibroblasts and keratinocytes release the matrix metalloproteinases (MMP) from specific storage granules into extracellular matrix (9).

Metalloproteinases play a fundamental role in all stages of healing, acting in tissue remodeling for degrading components of the extracellular matrix (ECM) and are able to act on the synthesis of collagen and other components. The imbalance between MMP and their inhibition propagates an inflammatory reaction, which slows healing and, thus, generates complications (10).

There are more than 25 metalloproteinases that can be grouped according to their substrate and structure: collagenases (MMP-1, 8 and 13), stromelysins (MMP-3, 7 and 10), gelatinases (MMP-2 and 9), matrilysins (MMP-7 and 26), membrane type MMPs (MMP-14, 15, 16, 17, 24) and other MMPs (Table-1) (10–12).

**Table 1:** Main matrix metalloproteinases, their enzymes and their substrates.

Metalloproteinase	Enzyme	Substrate
MMP-1	Collagenase 1	Collagen type 1, pro MMP-2
MMP-8	Collagenase 2	Proteoglycans
MMP-13	Collagenase 3	Collagen type 1
MMP-2	Gelatinase A	pro MMP-9, fibronectin, Collagen IV, V, VII and X; proteoglycans
MMP-9	Gelatinase B	Gelatin, fibronectin, elastin, collagen IV, V, VII, X denaturated Type I collagen
MMP-3	Stromelysin-1	Fibronectin, laminin, elastin, proteoglycan, collagen VI, V, IX, X, proMMPs −1,7,8,9,13
MMP-10	Stromelysin-2	Fibronectin, laminin, elastin, proteoglycan, collagen IV, V, IX, X
MMP-14	MT1-MMP	proMMP-2, −13, helical collagen
MMP-15	MT2-MMP	Surface transglutaminase
MMP-16	MT3-MMP	proMMP-2
MMP-17	MT4-MMP	Fibrin
MMP-7	Matrilysin	Fibronectin, elastin, collagen IV
MMP-12	Metalloelastase	Elastin
MMP-20	Enamelysin	Dental enamel matrix

The metalloproteinases analyzed in this study were MMP-2 and MMP-3, which have greater expression in the proliferation and maturation phases of healing, respectively. MMP-2 and MMP-9 are fundamental during angiogenesis, and they degrade collagen IV and other components of the extracellular matrix. MMP-3 is related to the maturation phase of wound healing (9).

## OBJECTIVE

To study the immunohistochemical quantification of IL-1 and TNF-α cytokines and MMP-2 and MMP-3 metalloproteinases in standard-weight (SW) versus lightweight (LW) meshes implanted in abdominal subcutaneous tissue of adult rats.

## MATERIALS AND METHODS

**Figure 1:**
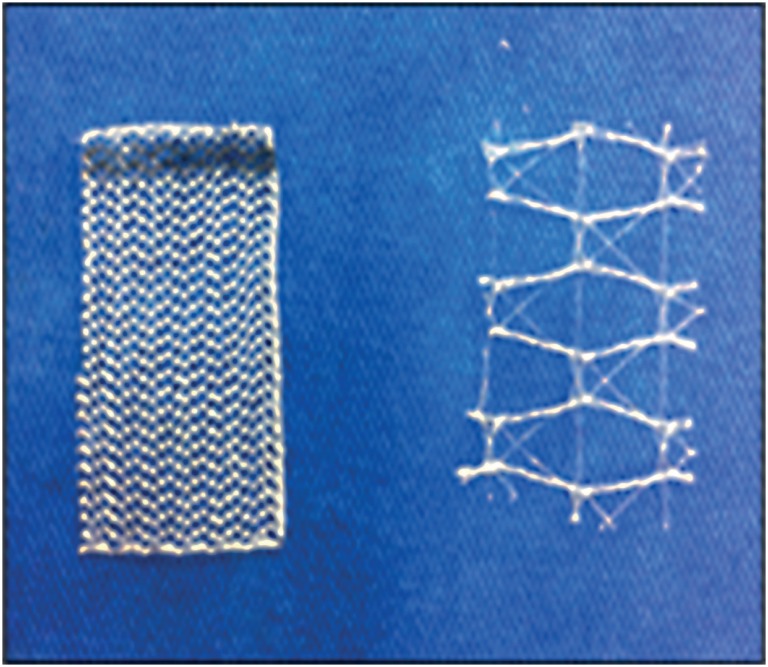
From left to right, standard-weight (72 g/m^2^) and lightweight (16 g/m^2^) polypropylene mesh.

The study used high-weight polypropylene mesh, referred to as standard-weight and low weight, respectively 72 g/m^2^ and 16 g/m^2^ (Figure-1).

**Figure 2:**
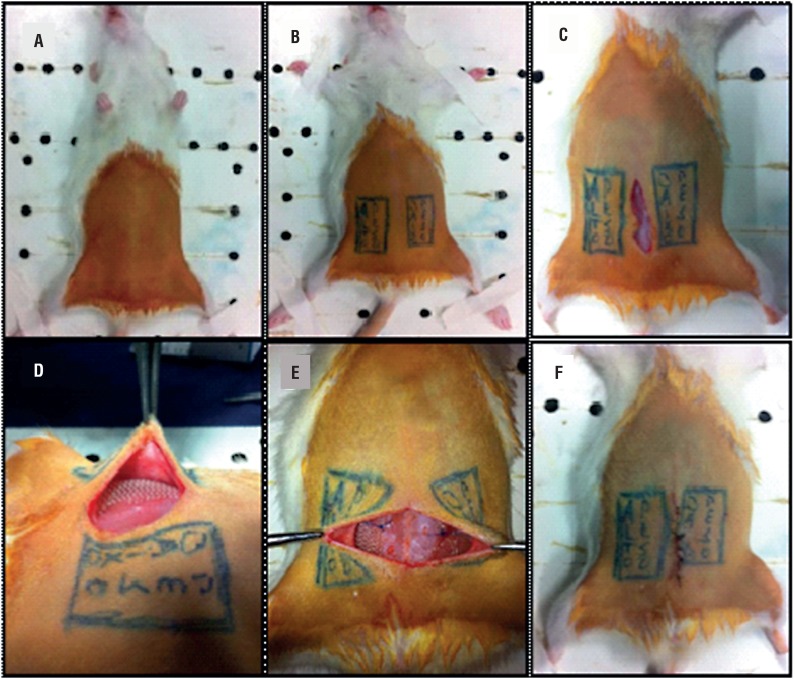
A) Positioning, B) Mesh implant scheme, C) 2.5 cm median incision, D) Mesh positioned upon aponeurosis, E) Mesh fixation with prolene 4.0, F) Skin suture.

Twenty female rats were submitted to the surgical procedure consisting of midline incision in the lower abdomen and subcutaneous dissection, where it was performed mesh implants, setting them side by side with polypropylene sutures 4.0 to a standardized procedure (SW on the right and LW on the left). Surgical scheme is presented at Figure-2.

They were then sorted into two groups regarding their time of euthanasia, 4 or 30 days.

The inflammatory reaction induced by the polypropylene mesh was then studied through immunohistochemical analysis quantifying the expressions of interleukin 1 (IL-1), tumor necrosis factor alpha (TNF-α) and matrix metalloproteinases 2 and 3 (MMP-2 and MMP-3).

The slides were analyzed by quantification of the inflammatory reaction surrounding the mesh, measuring the percentage of the area where there was expression of immunohistochemical reagents for IL-1 and TNF-α and metalloproteinases MMP-2 and MMP-3.

**Figure 3:**
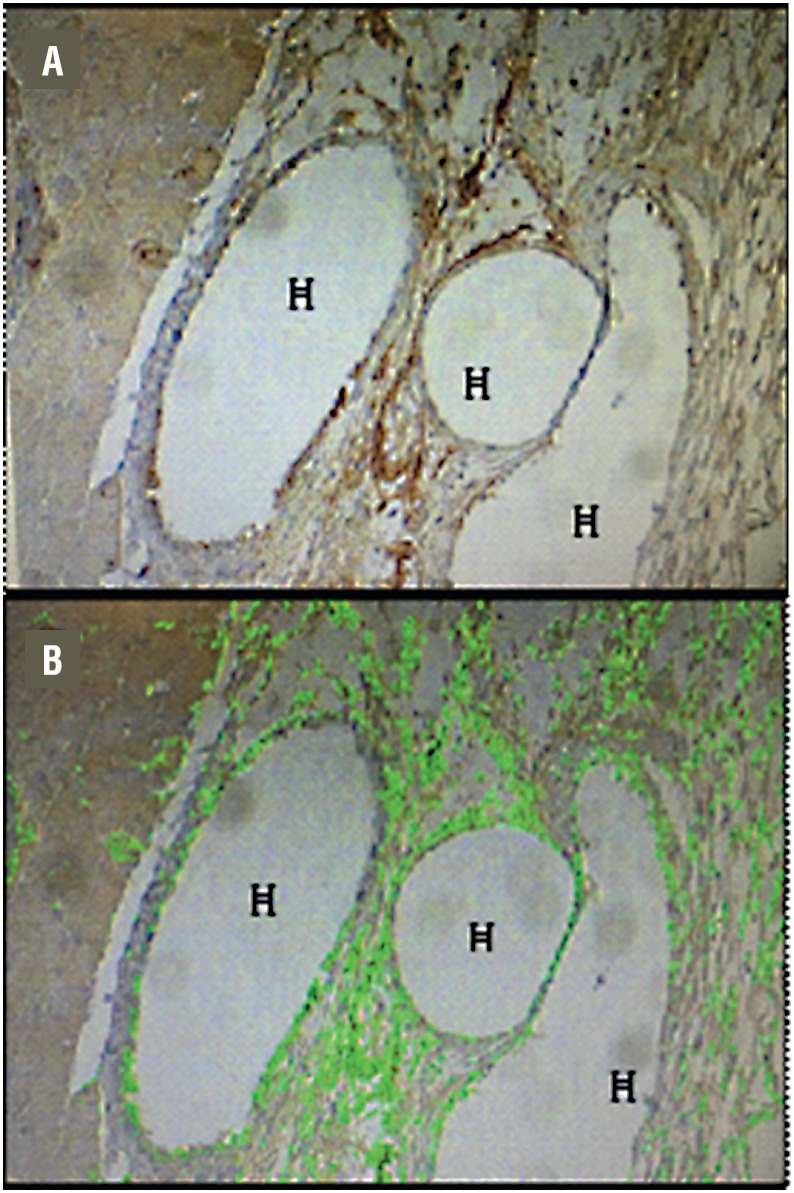
A) The brown color characterizes areas that are reactive to MMP3, B) Representation of Software Axiovision^®^ application that selects the reactive area in green and generates tables of the percentage area. *The gaps correspond to area occupied by mesh filaments.

Slide images were processed and stored with AxioVision^®^ software (Carl Zeiss Solutions). In Figure-3 it is shown an example of the software output image showing the immunoreactivity density and extension in slides marked for MMP3.

Statistical analysis was carried out from ANOVA for comparison between groups SW and LW, considering the euthanasia time of four or 30 days as well (13, 14).

## RESULTS

There were no deaths or complications during the post-implant period. No dehiscence or mesh exposure was observed either.

There was no statistically significant difference in the expression of inflammatory mediators IL-1 and TNF-α between the different types of mesh (lightweight or standard-weight). Nevertheless, there was an increase of IL-1 in the animals euthanized at the 30^th^ day (30 days >4 days) (p 0.0269).

No significant difference was also found regarding metalloproteinases MMP-2, between SW and LW meshes. However, there was an increase in the MMP-2 expression in the LW group compared to the group euthanized at the 30^th^ day. (LW: 30days >4 days) (p <0.001).

**Figure 4:**
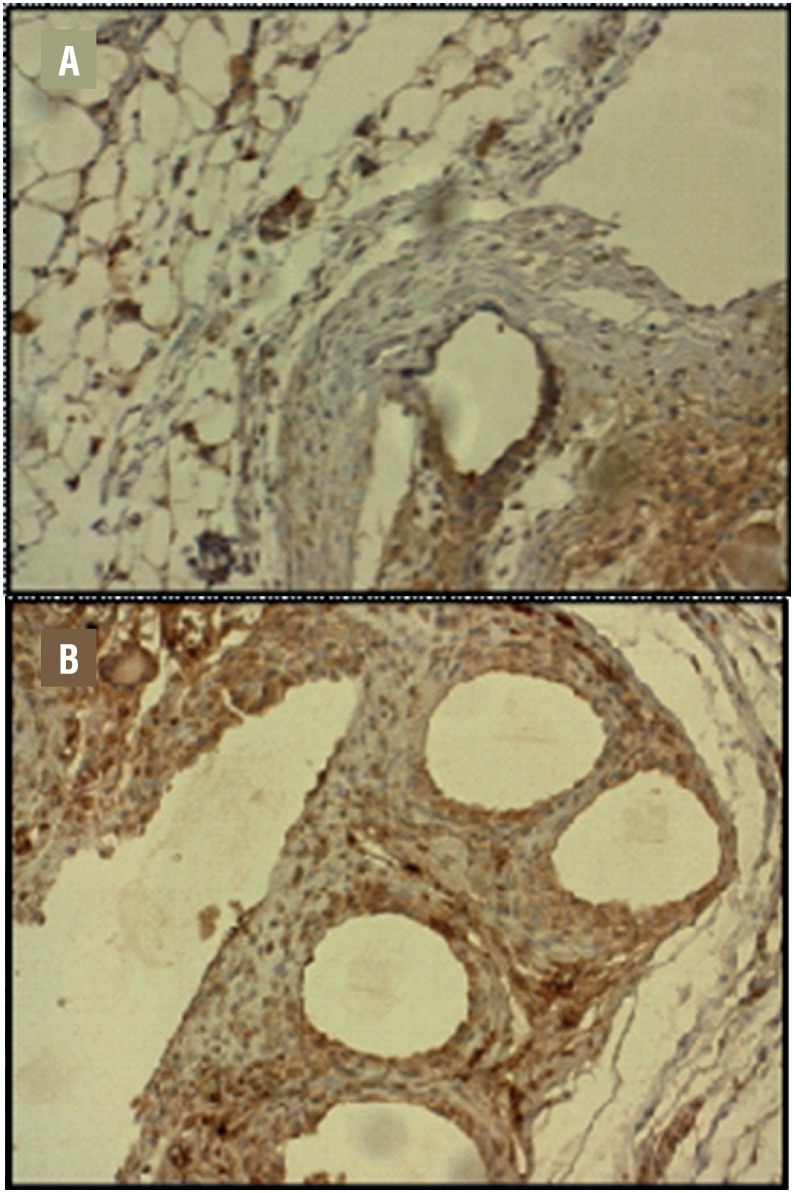
Example of MMP3 immunoreactivity (brown area) after 4 days (A) and 30 days (B) - (200x). Note a higher brown intensity and extension in B (30days >4 days).

MMP-3 presented similar expression in SW and LW meshes. In both groups its expression increased along time (30days >4 days) (p 0.02). Figure-4 exemplifies standard-weight mesh, in which MMP3 immunoreactivity was quantified. The brown area represents the MMP3 immunore-activity after 4 days (A) and 30 days (B) - (200x). Note the higher brown intensity and extension in B (30days >4 days) (Table-2).

**Table 2:** Immunohistochemistry analysis (mean percent area/SD) Immunohistochemical analysis of IL-1, TNF, MMP2 and MMP3 on euthanasia time and implant type, standard or low weight.

	IL-1*		TNF*		MMP2*		MMP3*	
	**SW (SD)**	**LW (SD)**	**SW (SD)**	**LW (SD)**	**SW (SD)**	**LW (SD)**	**SW (SD)**	**LW (SD)**
**4 Days**	10.28	11.92	12.22	11.22	14.56	15.18	10.68	11.81
	3.04	4.73	4.06	4.74	4.91	5.94	4.02	3.81
**30 Days**	14.83	14.15	13.31	11.64	15.1	20.87	15.77	16.34
	3.64	5.54	2.27	3.43	4.18	5.94	5.81	8.68
**p1**	0.0269		0.5509		0.2278		0.02	
**p2**	0.8347		0.2461		0.1115		0.8844	
**p3**	0.4157		0.7543		0.1854		0.8223	

* Mean percentage of the area marked by the immunohistochemistry reaction

**p1**= euthanasia time; **p2** = type of implant; **p3** = interaction euthanasia and type of implante; **SD** = Standard deviation; **SW** = standard-weight; **LW** = lightweight; **IL-1** = interleukin 1; **TNF** = tumor necrosis factor alpha; **MMP2** = matrix metalloproteinases 2; **MMP3** = matrix metalloproteinases 3

## DISCUSSION

The surgery, as well as the mesh implant, generates an inflammatory reaction with elease of cytokines and other inflammatory factors for tissue healing. The inflammatory reaction to biomaterial implant has been studied in order to better understand its integration to the host tissue and, consequently to develop biocompatible materials that may cause fewer complications. Thus, some studies describe the histological and molecular alterations after biomaterial implants.

The absence of significant difference in the quantification of these inflammatory markers, when comparing standard-weight and lightweight meshes, corroborate the findings from the literature, which do not relate the weight of the meshes with complications.

Utiyama et al. (15) concluded, in their study comparing heavyweight and lightweight meshes implanted in the abdomen of 30 rats who were euthanized after 21 days, that there was no difference in the inflammatory response (fibrosis, or infiltration of foreign body giant cells, macrophages, neutrophils or lymphocytes), mesh shrinkage, adherences or other complications.

Studies have pointed out that lightweight meshes are more malleable, tending to cause less chronic pain rates after abdominal hernia surgeries; nevertheless, they also show that there are no significant differences regarding complications or recurrences (16, 17).

Deffieux et al. have carried out a comparative study with 138 women who underwent repair of cystocele, and have not found any significant differences between lightweight and heavyweight meshes. Multifactorial analyses have evidenced age as a factor associated to erosion (18).

The present study has not found differences between standard-weight and lightweight meshes in the quantitative analysis of the interleukine-1 and tumor necrosis factor alpha. The difference in mesh's density did not influence the foreign body reaction, which lead us to assume that the occurrence of adverse events after implantation of PP prostheses could be influenced by other factors not exclusively related to the amount of implanted material.

In the initial phase of the inflammation, fibroblasts increase the production of extracellular metalloproteinases (MMP) which have an important role in tissue remodeling. Several studies have highlighted the role of MMP-2 in wound healing, from natural wounds to chronic wounds generated by biomaterial implants. The more intense the reaction to foreign body, the higher the MMP-2 gene expression, and with the stability of this reaction over time, there would be a reduction of MMP-2 surrounding the mesh (19).

The fact that in the present study there was not a significant expression of metalloproteinases MMP-2 in the groups where the rats were euthanized four days after the implant meets the findings of Iba et al. who have observed that, in rodents, the levels of MMP-2 and MMP-9 are increased 10 to 15 days after the wound, coinciding with angiogenesis activation (20).

MMP-3 is an enzyme related to the remodeling phase, thus, the increase of its expression in later phases was confirmed in the study (30 days >4days). However, there was no significant difference between groups SW and LW.

Another study in 49 Rhesus monkeys found that in the heavyweight less porous and less malleable mesh implants, the degradation of vaginal collagen and elastin has exceeded synthesis, probably as a result of the increased activity of the MMPs, resulting in structurally compromised tissue (21).

Jansen et al. implanted monofilament heavyweight polypropylene meshes, lightweight multifilament and absorbable meshes in 72 mice, and did not find differences in the number of macrophages, fibroblasts and MMP-2, concluding that these parameters depend on the location and on the types of cells rather than on mesh structure (22).

Histological and molecular studies on inflammatory reactions to biomaterials can lead to the better understanding of the interaction of these biomaterials with the host tissue, helping to clarify which parameters would improve tissue integration, and thus reduce complication rates. However, conclusions on which cells and markers must be assessed are not yet conclusive.

This study has limitations, such as not using vaginal site, no counting of differential inflammatory cells, absence of supply division of collagen type I and III, no quantification of immunostaining of anti-inflammatory cytokines such as TGF-β and quantification of TIMP (metalloproteinase inhibitors) and no quantification of the vessels (angiogenesis).

## CONCLUSIONS

Standard-weight and lightweight mesh implants in subcutaneous abdominal tissue of female adult rats induced similar histochemical reactivity of the pro-inflammatory cytokines IL-1 and TNF-α and metalloproteinases MMP-2 and MMP-3. We infer that other pathways should be studied to justify the differences between standard-weight and lightweight mesh implants in clinical setting.

## ABBREVIATIONS

POP: pelvic organ prolapse
FDA: Food and Drug Administration
IL-1: Interleukin-1
TGF-β: Growth factor beta
TNF-α: tumor necrosis factor alpha
MMP: matrix metalloproteinases
PP: polypropilene
TIMP: metalloproteinase inhibitors
HW: heavyweight
LW: lightweight
SW: standard-weight
DPI: dots per inch
